# Fecal calprotectin and platelet count predict histologic disease activity in pediatric ulcerative colitis: results from a projection-predictive feature selection

**DOI:** 10.1007/s00431-024-05554-y

**Published:** 2024-05-06

**Authors:** B. Schiller, E. Wirthgen, F. Weber, S. Schiller, M. Radke, M. Claßen, J. Däbritz, CEDATA-GPGE Study Group, S. Buderus, P. Bufler, J. Däbritz, S. Dammann, J. de Laffolie, M. Friedt, A. Hauer, K. M. Keller, A. Krahl, M. Laaß, T. Lang, C. Posovszky, B. Rodeck, S. Trenkel

**Affiliations:** 1 Department of Pediatrics, Rostock University Medical Center, Rostock, Germany; 2 Institute for Biostatistics and Informatics in Medicine and Ageing Research, Rostock University Medical Center, Rostock, Germany; 3 Department of Pediatrics, Greifswald University Medical Center, Greifswald, Germany; 4 Department of Pediatrics and Adolescent Medicine, Erlangen University Medical Center, Erlangen, Germany

**Keywords:** Inflammatory bowel disease, Ulcerative colitis, Histopathology, Calprotectin, Platelets, Bayesian

## Abstract

Especially for pediatric patients, proxies of mucosal inflammation are needed. The Pediatric Ulcerative Colitis Activity Index (PUCAI) has been established to predict clinical and endoscopic disease activity. However, histologic inflammation might persist. We applied a special variable selection technique to predict histologic healing in pediatric ulcerative colitis (UC) as parsimoniously (but still as precisely) as possible. The retrospective analysis included data from two study cohorts, comprising 91 visits from 59 pediatric patients with UC. A Bayesian ordinal regression model was used in combination with a projection-predictive feature selection (PPFS) to identify a minimal subset of clinical and laboratory parameters sufficient for the prediction of histologic disease activity. Following the PPFS, CEDATA-GPGE patient registry data were analyzed to investigate the relevance of the selected predictors in relation to PUCAI and Physician Global Assessment (PGA) in up to 6697 patient visits. Fecal calprotectin (FC) and platelet count were identified as the minimal subset of predictors sufficient for prediction of histologic disease activity in pediatric UC. FC and platelet count also appeared to be associated with increasing disease activity as measured by PUCAI and PGA in the CEDATA-GPGE registry. Based on the selected model, predictions can be performed with a Shiny web app.

*  Conclusion*: Our statistical approach constitutes a reproducible and objective tool to select a minimal subset of the most informative parameters to predict histologic inflammation in pediatric UC. A Shiny app shows how physicians may predict the histologic activity in a user-friendly way using FC and platelet count. To generalize the findings, further prospective studies will be needed.

**Table:** 

**What is Known:** *• Histologic healing is a major endpoint in the therapy of ulcerative colitis (UC).* *• The PUCAI score has been established to predict disease activity in pediatric UC but is not suitable for the prediction of histologic healing.*	
**What is New:** *• Our Bayesian ordinal regression model in combination with a projection-predictive feature selection is a reproducible and objective tool to select the minimal subset of clinical and laboratory parameters to predict histologic inflammation in pediatric UC.* *• Histologic inflammation in pediatric UC can be non-invasively predicted based on the combination of fecal calprotectin levels and platelet count.*

**Supplementary Information:**

The online version contains supplementary material available at 10.1007/s00431-024-05554-y.

## Introduction

Ulcerative colitis (UC) is an inflammatory bowel disease (IBD) characterized by chronic inflammation of the colon and a rising incidence/prevalence worldwide in children and adolescents [1]. Assessment of disease activity is critical for clinical management, therapeutic decisions, and monitoring of treatment targets [2]. Endoscopic examination with tissue sampling provides a definitive assessment of the current inflammatory activity. Absence of endoscopic inflammation such as friability, blood, erosions, and ulcers in the visualized intestinal mucosa indicates mucosal healing [3, 4]. Histologic assessment of tissue biopsies without evidence of inflammatory cell infiltrates, in particular neutrophils, indicates that histologic healing is achieved [5, 6]. The Mayo endoscopic subscore is useful for determining mucosal healing [7, 8], while the Nancy index is valuable for the assessment of histologic disease activity in UC [9]. In addition to endoscopic remission, histologic remission has become an emerging treatment target in UC clinical trials [10] and is recommended by the European Crohn’s and Colitis Organisation [11].

Both endoscopy and histologic assessment are invasive medical procedures. Especially in pediatrics, hospitalization is often necessary to ensure an adequate (retro-/antegrade) bowel cleansing and to provide sedation or anesthesia for endoscopies and biopsies in a child-friendly environment and with sufficient quality. Bowel cleansing, analgesic sedation, and endoscopy with biopsies are not only associated with significant risks and complications for the patient, but they also tie up relevant resources of the healthcare system [12, 13]. Therefore, non-invasive measuring tools are required to minimize the invasiveness, the discomfort, and the potential complications associated with these procedures. For pediatric UC, the Pediatric Ulcerative Colitis Activity Index (PUCAI) was developed to non-invasively predict the disease activity [14]. It was developed using the Delphi group technique and correlated strongly with colonoscopy, Physician Global Assessment (PGA), and Mayo score [7, 14]. However, the PUCAI was not designed to reflect histologic inflammation and therefore cannot be used to predict histologic remission/healing, which is determined as one of the therapeutic targets in clinical practice and in current studies [15, 16]. One reason for the relevance of histologic assessment relates to the fact that patients with mucosal healing still have histologic inflammatory activity in up to 30% of cases [17]. The absence of histologic healing is associated with an increased risk for clinical relapse, hospitalization, subsequent dysplasia, or surgery [5, 18–21]. Hence, in clinical practice, endoscopy with tissue sampling is often performed before essential therapeutic decisions to include histologic inflammatory activity as an indicator of remission depth in decision-making [22–24].

Accordingly, the aim of this study is to identify a minimal subset of non-invasive parameters that reflect the histologic disease activity in pediatric UC sufficiently enough and to present a feasible method how to predict histologic disease activity in daily clinical practice. To this end, we applied a Bayesian ordinal regression model and a projection-predictive feature selection, the rationale of which has already been explained in detail previously [25].

## Patients and methods

The data used in this study were collected between September 2015 and July 2023 at two German pediatric IBD centers, the Rostock University Medical Center (Department of Pediatrics) and the Klinikum Westbrandenburg (Department of Pediatrics). The ethics committees of both centers approved this study under registration numbers A 2020–0161 (Rostock) and AS 73(bB)/2020 (Potsdam). In addition, data from the CEDATA-GPGE registry were analyzed to compare predictors of histologic inflammatory activity identified in the aforementioned study population with data from the CEDATA-GPGE registry collected in Germany and Austria between July 2014 and December 2022. CEDATA-GPGE registry was approved by the ethics committee of the Giessen University Medical Center (Germany) under registration number 07/11.

### Patients

This study included data from 91 visits of 59 children and adolescents (< 18 years) with a confirmed diagnosis of UC based on international criteria for the diagnosis of IBD in children and adolescents [26]. Inclusion criteria for this study were visits in which a complete ileocolonoscopy with histologic examination was performed. In addition, it was a prerequisite that patients underwent a fecal calprotectin test within the last 30 days and a laboratory test within the last 14 days prior to the endoscopy. Patients with fever > 38 °C and signs of infection on physical examination on the day of endoscopy or admission to the clinic, a history of infection, and children and adolescents with suspected infectious gastroenteritis by stool polymerase chain reaction (*Campylobacter*/*Salmonella*/*Shigella*/*Vibrio*/*Aeromonas spec.*, *Yersinia enterocolitica*, *Clostridioides difficile*, norovirus, rotavirus, adenovirus, astrovirus, and sapovirus) were excluded from the study.

The data analyzed from CEDATA-GPGE cohort consisted of children and adolescents under the age of 18 years with a confirmed diagnosis of ulcerative colitis. To obtain an in-depth understanding of the data structure and historical background of the CEDATA-GPGE registry, please refer to Buderus et al. [27] and Leiz et al. [28].

### Assessment of histologic and endoscopic inflammation

Histologic inflammation in all study participants was routinely assessed and documented by trained pathologists using formalin-fixed biopsies. Based on the histologic findings and the pathologist’s assessment, histologic inflammation was retrospectively assigned into 5 categories based on the Nancy index [9] as recommended by a consensus expert panel [11]. Grade 0 was defined as the absence of histologic inflammation. Grade 1 was characterized by chronic infiltrates without evidence of acute inflammatory infiltrates, mildly active disease (grade 2) by mild acute inflammatory cell infiltrates, and moderately active disease (grade 3) by moderate to severe acute inflammatory cell infiltrates. Grade 4 (severely active disease) was determined by the presence of ulceration. Due to the low frequency of grades 0 and 1, and the study objective to distinguish between active and inactive inflammatory activity, these categories were combined for data analysis and referred to as remission/non-active.

Mucosal appearance at endoscopy was retrospectively assigned to the Mayo endoscopic subscore [7] based on detailed written examination reports collected and documented by trained pediatric gastroenterologists. The Mayo endoscopic subscore is classified as normal or inactive disease, mild disease (erythema, reduced vascular pattern, and mild friability), moderate disease (marked erythema, absent vascular pattern, friability, and erosions), or severe disease (spontaneous bleeding, ulceration).

For statistical analysis, the histologically and endoscopically most severely inflamed areas were considered, regardless of location in the colon or terminal ileum.

### Candidate predictors of histologic inflammation

The statistical analysis of candidate predictors included parameters derived from the patient’s medical history, the physician’s physical examination, laboratory findings (blood/stool), and demographic information. An overview of all candidate predictors is presented in Supplementary Table S1. Other parameters initially collected (serum albumin, erythrocyte sedimentation rate, alanine-aminotransferase, gamma-glutamyltransferase, pancreatic lipase, creatinine, height gain, Tanner stages, and appetite) could not be included in the statistical analysis due to a high number of missing values (due to ambiguous or missing documentation).

### Statistics

For our statistical analysis, we followed the approach of Wirthgen and Weber et al. [25]. Briefly, using the R [29] package brms [30–33] which is based on Stan [34], we fitted a Bayesian ordinal regression model to our data, and then, using the R package projpred [35–37], we performed a projection-predictive feature selection (PPFS) based on this reference model. The reference model’s outcome was the 4-category histologic inflammation as described in the “Assessment of histologic and endoscopic inflammation” section and its predictors were the candidate predictors listed in Supplementary Table S1, but standardized and partially log-transformed as described previously [25]. We emphasize the inclusion of group-level (“random”) intercepts for the patient identifiers in the reference model to account for correlation of visits coming from the same patient. For the prior, we used brms’s default priors for all model parameters, except for the population-level regression coefficients, for which we used a regularized horseshoe prior [38]. Since we performed a complete-case analysis, the dataset used for this ordinal regression model (and hence also for the PPFS) had 85 observations (patient visits). The aim of the PPFS can be summarized as selecting a subset of the candidate predictors that is as small as possible (hence a minimal subset) but still achieves a predictive performance that is as good as possible. In order to facilitate the way predictions are made based on the selected submodel resulting from the PPFS, we developed a Shiny [39] web application.

Following the PPFS, we analyzed data from the CEDATA-GPGE registry to investigate the relevance of the selected predictors in relation to PUCAI and PGA using the Spearman correlation *r*_s_ (with 95% confidence interval, 95% CI) and receiver operating characteristic (ROC) curve analysis implemented in SPSS version 29.0 for Macintosh [40]. Area under the curve (AUC) results with values > 0.7 are considered fair, > 0.8 considerable, and > 0.9 excellent [41]. The Youden Index was used to find an optimal cut-off point.

## Results

### Characteristics of the study cohort and distribution of histologic/endoscopic inflammation

**Fig. 1:**
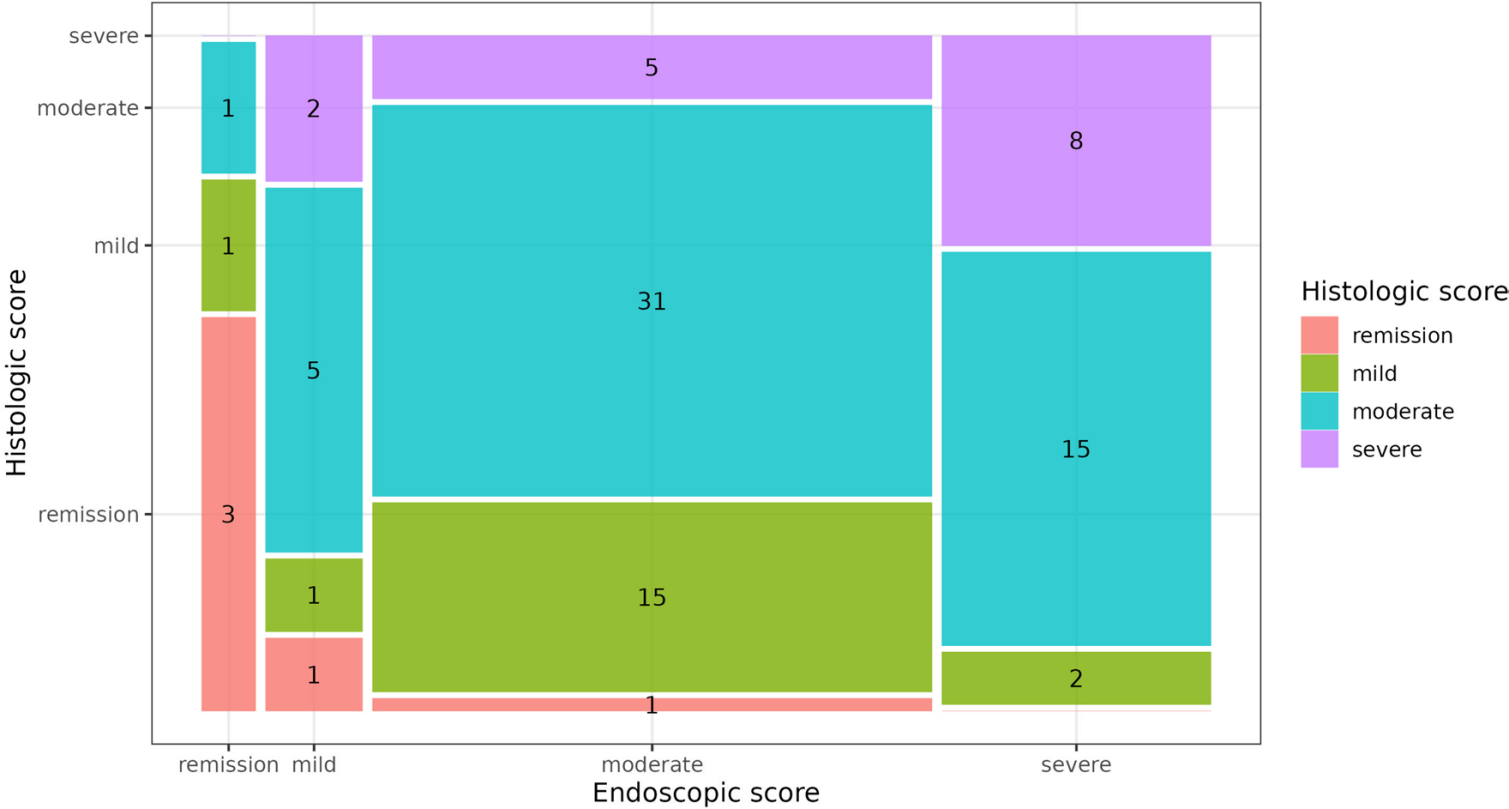
Frequency of the endoscopic and histologic score categories. This mosaic plot is a graphical display of a contingency table. Numbers indicate absolute frequencies (numbers of visits). Empty category combinations are indicated by a thin line (lacking the space for the value “0”)

The mean age of the 35 female and 24 male children and adolescents was 13.8 years (standard deviation: 3.4 years; minimum: 2.5 years; maximum: 17.9 years). A complete overview of the general patient characteristics is given in Supplementary Table S2. Out of 91 visits analyzed descriptively, the majority of children and adolescents (57.1%) had moderate histologic inflammation (grade 3). Only in five visits (5.5%) histologic remission or no acute inflammatory activity occurred (grade 0). In the remainder of the visits (37.4%), histologic disease activity was equally distributed between mild and severe inflammation (grades 2 and 4). The comparison of endoscopic and histologic scores revealed a discrepancy between histologic and endoscopic inflammation (Fig. 1). Histologic remission was observed in only three of the five visits with endoscopic remission, while in two visits, mild and moderate histologic inflammatory activity was detectable. Even in visits with mild, moderate, and severe endoscopic inflammation, there was no concordance with histologic inflammatory activity in at least 40%.

### Distribution of the candidate predictors across the histologic score categories

**Fig. 2:**
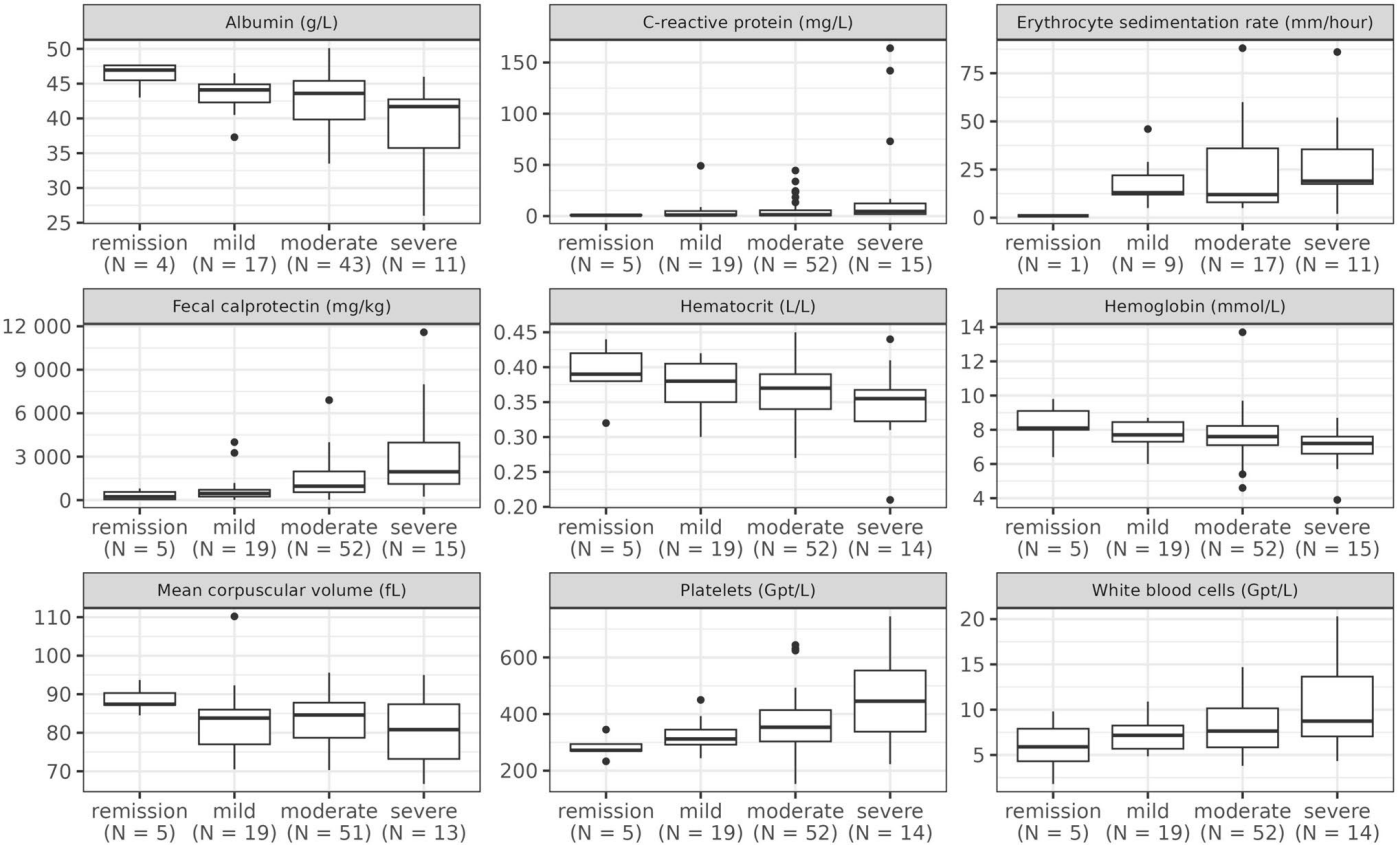
Distribution of laboratory parameters across the histologic inflammation categories. The boxplots comprise the lower hinge, median, and upper hinge. Those boxplots also visualize albumin and the erythrocyte sedimentation rate, which had to be excluded as candidate predictors due to a high number of missing values

The median values of albumin, hematocrit, and hemoglobin decreased with rising grade of histologic inflammation, while those of C-reactive protein (CRP), fecal calprotectin (FC), platelets, and white blood cells increased. The empirical distribution of laboratory parameters across the histologic inflammation score is presented in Fig. 2. In contrast to laboratory findings, categorical candidate predictors have no continuous values but comprise two or more categories. A summary of the distribution of the categorical candidate predictors is provided in Supplementary Table S3.

### FC and platelet count as predictors of histologic inflammation

**Fig. 3:**
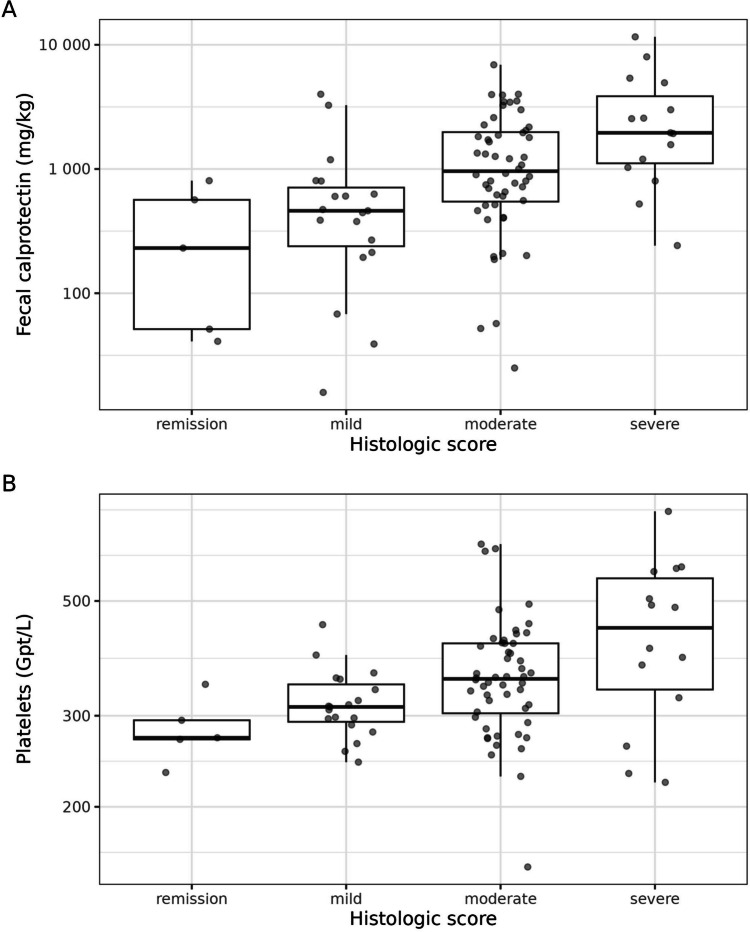
Distribution of fecal calprotectin (**A**) and platelets (**B**) across the histologic score categories. The boxes of the boxplots consist of lower hinge, median, and upper hinge. The *y*-axes are log-scaled. These data are also shown at lower resolution in Fig. 2. As FC and platelet count are the two identified predictors, the boxplots in Fig. 3 show their distribution across the histologic categories even more clearly, at higher resolution and with log-scaled *y*-axes

**Fig. 4:**
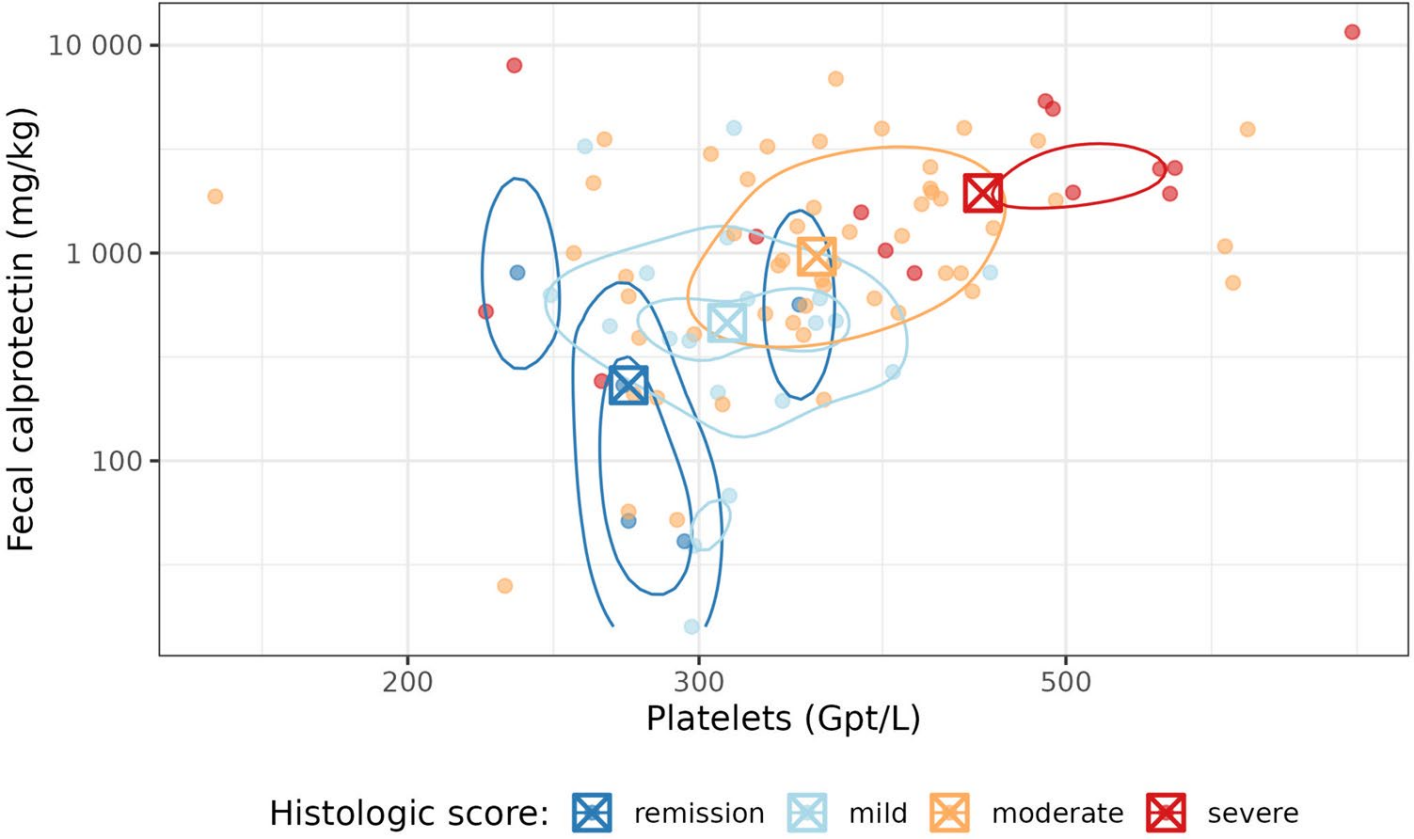
Joint distribution of fecal calprotectin and platelet count, together with their association with the histologic score. The contour lines illustrate two-dimensional kernel density estimates. The boxed crosses indicate the category-wise medians of fecal calprotectin and platelet count. Both axes are log-scaled

**Fig. 5:**
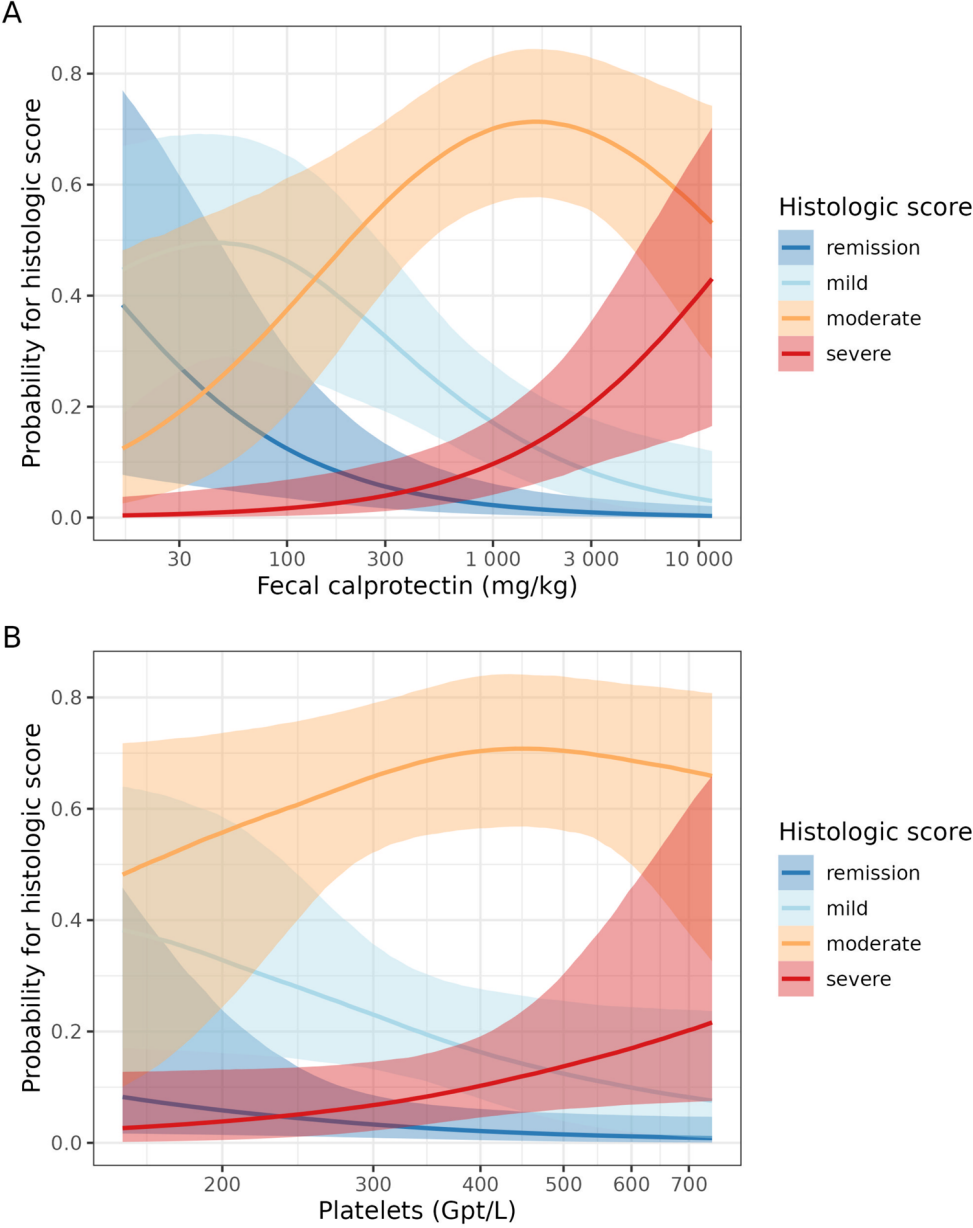
Estimated projected effect of fecal calprotectin (**A**) and platelet count (**B**) on the histologic score (conditional-effects plots from the selected histologic submodel, SHSM). Note that these plots should not be interpreted as showing the isolated effects of fecal calprotectin and platelet count since they are based on the projected posterior. These plots condition on the mean (standardized and log-transformed) platelet count and fecal calprotectin (for **A** and **B**, respectively). The semi-transparent bands indicate 95% uncertainty (projected posterior) intervals. The *x*-axes are log-scaled

As illustrated by the size selection plot for the PPFS (Supplementary Fig. S1), already two predictors achieved a sufficient predictive performance. A third predictor provided no improvement with respect to predictive performance. The PPFS’s full-data predictor ranking revealed that FC and platelet count are the two parameters that should be selected (Supplementary Table S4). Hence, to obtain our selected histologic submodel (SHSM), the reference model was projected again (this time with a higher “resolution”) onto the submodel comprising FC and platelet count. The separate plots of the empirical distribution of FC and platelet count across the histologic inflammation categories revealed that with rising FC and platelet count, the likelihood of more severe histologic inflammatory activity increased (Fig. 3). The clustered empirical distribution of FC and platelet count as a function of histologic inflammatory activity supported this assumption, although the scatter of individual values is rather wide (Fig. 4). Figure 5 illustrates the projected effects of FC and platelet count on the histopathologic score using conditional-effects plots derived from the SHSM. These plots are not intended to represent the isolated effects of FC and platelets since they are based on the projected posterior. Derived from the projected posterior are also the semitransparent bands which represent 95% uncertainty intervals.

### Feasible non-invasive prediction of histologic inflammatory activity

The SHSM can be used to predict histologic inflammation in a user-friendly and simple manner by the help of a Shiny web application [39] that accepts values for the selected predictors (FC and platelet count) as input. An example of a possible Shiny application (https://umrukj.shinyapps.io/shsm/) is shown in Supplementary Fig. S2. In contrast to the existing PUCAI, this app does not return a single value on an ordinal scale, but rather the predictive probability for the presence of each possible severity level (inactive, mild, moderate, and severe), thus providing a more complete picture than a single value.

### Distribution of FC and platelet count across the PUCAI and PGA categories based on data from the CEDATA-GPGE registry

**Fig. 6:**
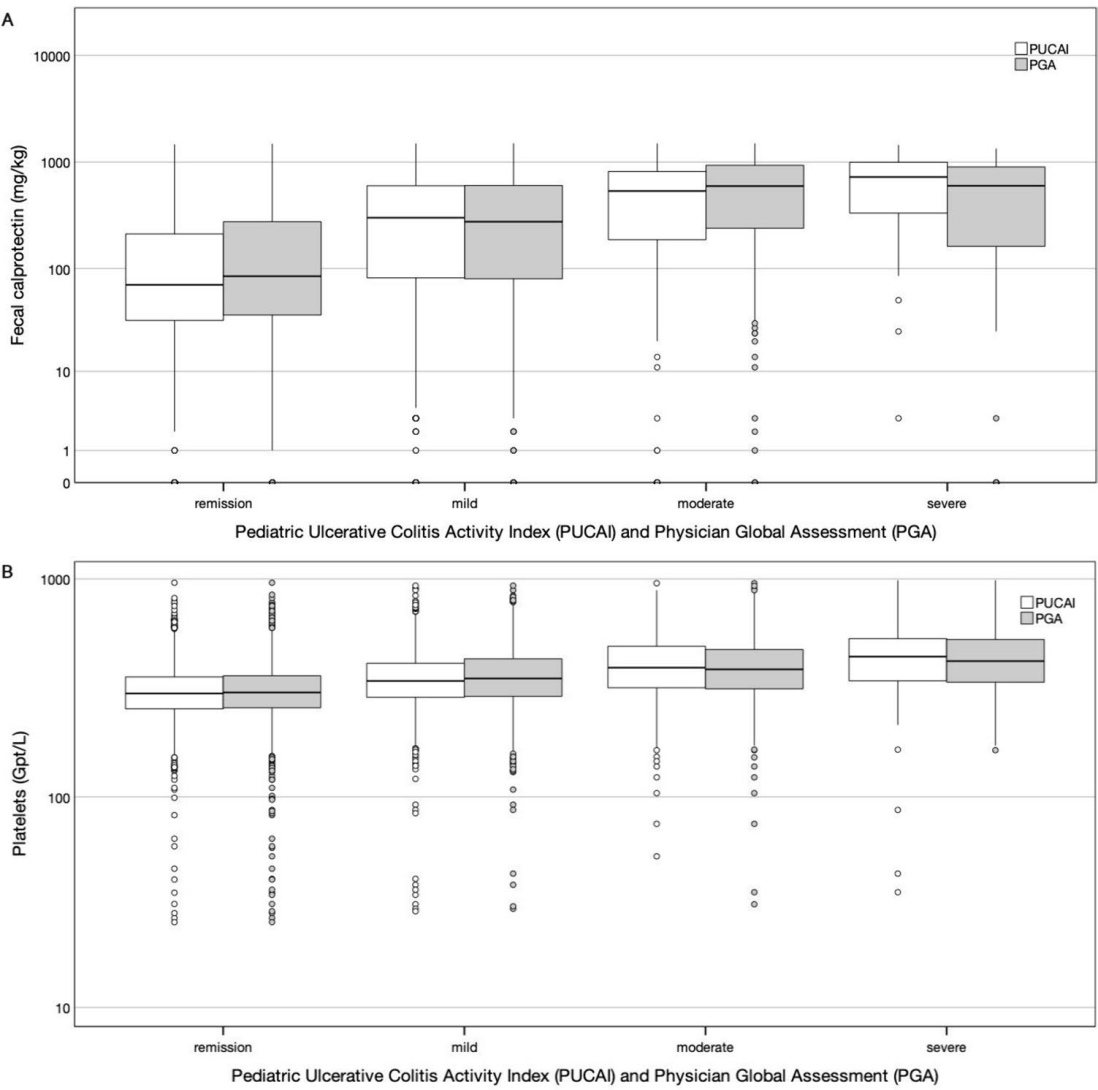
Distribution of fecal calprotectin (**A**) and platelet count (**B**) across the PUCAI and the PGA categories based on data from the CEDATA-GPGE registry. PGA is based on subjective physician assessment. PUCAI categories are classified as remission (< 10 points), mild activity (10 to 39 points), moderate activity (40 to 64 points), and severe activity (65 to 85 points). The boxes of the boxplots consist of lower hinge, median, and upper hinge. The *y*-axes are log-scaled

Based on data from the CEDATA-GPGE registry, the distribution of FC and platelet count across the PUCAI and PGA categories is shown in Fig. 6. A total of 2873 data pairs were obtained for the correlation between FC and PUCAI, 2981 for FC and PGA, 6697 for platelet count and PUCAI, and 6075 for platelet count and PGA. Spearman correlation indicated a positive association of platelet count with disease severity measured by both, PUCAI and PGA (PUCAI: *r*_s_ = 0.30, 95% CI = [0.27, 0.32]; PGA: *r*_s_ = 0.33, 95% CI = [0.31, 0.36]). For FC, a positive correlation with both, PUCAI and PGA, was observed as well (PUCAI: *r*_s_ = 0.33, 95% CI = [0.30, 0.37]; PGA: *r*_s_ = 0.40, 95% CI = [0.36, 0.43]).

For PGA (remission/mild vs. moderate/severe), the AUC was moderate with values of 0.711 for platelets and 0.740 for fecal calprotectin (Supplementary Fig. S3A). The optimal cut-off for platelets was 380.5 Gpt/L with a sensitivity of 53.7% and specificity of 79.8% (Youden Index = 0.337). For fecal calprotectin, the optimal cut-off was 275.5 mg/kg (sensitivity = 71.7%, specificity = 68.4%, and Youden Index = 0.461).

The AUC for PUCAI (remission/mild vs. moderate/severe) was moderate with values of 0.706 for platelets and 0.742 for fecal calprotectin, respectively (Supplementary Fig. S3B). Cut-off for platelets was 347.5 Gpt/L (sensitivity = 64.2%, specificity = 68.8%, and Youden Index = 0.33) and 335.5 mg/kg for fecal calprotectin (sensitivity = 66.9%, specificity = 72.5%, and Youden Index = 0.394).

## Discussion

Our study revealed that histologic inflammatory activity in pediatric UC can be predicted parsimoniously by the combination of FC and platelet count. Other investigated laboratory parameters or subjective information obtained from medical history did not improve the prediction of histologic inflammatory activity.

For comprehensive disease management and improvement of patient outcomes, the strategy of combining treatment targets (clinical, endoscopic, and histologic remission, respectively histologic healing) to achieve a deeper level of healing is recommended in UC [42]. In this study, we focused on the prediction of histologic healing because histologic inflammatory activity is frequently inconsistent with endoscopic inflammatory activity in approximately 30% of the cases [17], and to date, no model has been published that provides a convenient and non-invasive prediction of histologic inflammatory activity in pediatric UC. In our study, the comparison between histologic and endoscopic inflammation scores also revealed discrepancies in the assumed severity of inflammation depending on which score is used. In fact, histo-endoscopically inactive disease is associated with reduced IBD disability as measured by patient-reported outcome measurements; however, patient-reported outcome measurements for disability and clinical disease activity cannot completely replace histo-endoscopic findings [43]. In addition, the assessment of histologic disease activity assumes increasing relevance [44], as histologically active disease despite endoscopic remission increases the risk of clinical relapse, hospitalization, subsequent dysplasia, or surgery [5, 18–21].

Moreover, higher grades of histologic inflammatory activity were associated with a higher frequency of and a shorter time to UC progression [45]. Recent evidence suggests that neutrophil mucosal infiltration might be a key discriminator between active disease and remission [46–48] and complete resolution of neutrophil-associated acute inflammation as marker of histologic remission is of importance as a target for treatment of UC [49]. Our model, including FC and platelet count, may serve as a proxy for histologic healing and contribute to improve the management of pediatric UC in combination with clinical findings.

Our results reveal a high predictive value for the platelet count, confirming numerous studies describing a positive correlation of platelets with inflammatory activity in UC [50–52]. Our additional analyses of data from the CEDATA-GPGE registry also revealed an increasing platelet count with increasing inflammatory activity, reflected by PUCAI and PGA. All described findings confirm the crucial role of platelets in inflammation including IBD [52–55]. The demonstrated relevance of platelet count as a biomarker in UC may also improve the informative value of other markers when combined with platelet count, as it has been shown in the platelet-to-lymphocyte ratio [56] or neutrophil-to-platelet ratio [57] in predicting disease activity. In this study, we showed that platelet count also increases with increasing histologic inflammatory activity in pediatric UC confirming its significance as a biomarker for mucosal inflammation. According to the described interference of activated platelets with leukocyte trafficking and effector functions of neutrophils and macrophages, our results underpin the predictive value of platelets, in particular for histologic inflammation, characterized by the presence of leukocytes in the mucosa.

FC reflects neutrophil migration across the inflamed gastrointestinal mucosa into the gastrointestinal tract [58] and correlates well with endoscopic [59] and histologic inflammatory activity in UC [60, 61]. Complementary analysis of the CEDATA-GPGE registry emphasized the role of fecal calprotectin in assessing disease activity by confirming the positive association of FC with PUCAI and PGA.

An important requirement for the implementation of disease activity indices in clinical practice is a simple and time-saving usability in everyday clinical practice. Therefore, we demonstrated the simple application of the SHSM using FC and platelet count with a demo version of a Shiny app (https://umrukj.shinyapps.io/shsm/). However, it should be noted that, before using it in clinical practice, our approach needs to be repeated in a prospective study with additional data to improve the reliability of the SHSM.

This study is also constrained by a limited number of patients, in particular, a low number of patients in remission. Since pediatric patients without symptoms typically underwent no endoscopy to avoid the discomfort of hospitalization as well as the potential risks and complications of endoscopy and analgesia. To improve the predictive accuracy and the reliability of the Shiny app and to validate the results, another prospective study with a larger and external cohort is required, especially including more patients in remission. For further objectification of the histologic inflammation assessment, the evaluation of biopsies using artificial intelligence may be considered to reduce inter-observer variances [62].

Moreover, we emphasize that our methodology allows future studies to include predictors which have not yet been considered due to a high number of missing values or due to ambiguous or missing documentation (height gain, serum albumin, erythrocyte sedimentation rate, alanine-aminotransferase, gamma-glutamyltransferase, pancreas lipase, creatinine, iron metabolism parameters, height gain, and Tanner stages) or entirely newly collected parameters. Moreover, due to the proven relevance of neutrophils [57] and lymphocytes [56], a differential blood count should be included in further investigations. It will then become clear whether the inclusion of other predictors in the statistical model will lead to an improvement in predictive quality or whether FC and platelet count are the most meaningful parameters.

Additional statistical aspects are discussed in the electronic supplementary material.

In conclusion, we demonstrated that the combination of FC and platelet count is suitable for non-invasive prediction of histologic inflammatory activity in pediatric UC. Based on the results, this study might pave the way for the establishment of a non-invasive score to assess histologic healing in pediatric UC and to improve quality of care for children and adolescent living with IBD. The easy-to-use prediction can be performed using a Shiny app.

### Supplementary Information

Below is the link to the electronic supplementary material.

Supplementary file1 (PDF 656 KB)

## Data Availability

For all analyses except those based on the CEDATA-GPGE registry data, the datasets and source code can be found in the Open Science Framework (OSF) at 10.17605/OSF.IO/G8HF5. The CEDATA-GPGE raw data of this article will be made available by the authors, upon reasonable request.

## Abbreviations

CRP: C-reactive protein
FC: Fecal calprotectin
IBD: Inflammatory bowel disease
PGA: Physician Global Assessment
PPFS: Projection-predictive feature selection
PUCAI: Pediatric Ulcerative Colitis Activity Index
SHSM: Selected histologic submodel
UC: Ulcerative colitis
