# Supplementary material for: Fecal calprotectin and platelet count predict histologic disease activity in pediatric ulcerative colitis: results from a projection-predictive feature selection
Source: Eur J Pediatr. 2024 May 6;183(8):3277–88. doi: 10.1007/s00431-024-05554-y (PMC11263432; doi:10.1007/s00431-024-05554-y)
Supplement: Supplementary file 1 — Supplementary file1 (PDF 656 KB) [file 431_2024_5554_MOESM1_ESM.pdf]

ELECTRONIC SUPPLEMENTARY MATERIAL

for

„Fecal calprotectin and platelet count predict histologic disease activity in pediatric ulcerative colitis: Results from a projection-predictive feature selection“

Schiller B[1*], Wirthgen E[1*], Weber F[2], Schiller S[1,3], Radke M[1], Claßen M[4], Däbritz J[1,3];
CEDATA-GPGE Study Group

Affiliations
[1]Department of Pediatrics, Rostock University Medical Center, Rostock, Germany;
[2]Institute for Biostatistics and Informatics in Medicine and Ageing Research, Rostock University Medical Center, Rostock, Germany;
[3]Department of Pediatrics, Greifswald University Medical Center, Greifswald, Germany
[4]Department of Pediatrics and Adolescent Medicine, Erlangen University Medical Center, Erlangen, Germany
*These authors contributed equally to this work.

Corresponding author
Prof. Dr. Jan Däbritz, M.D.
Greifswald University Medical Center
Department of Pediatrics
Ferdinand-Sauerbruch-Str.1
D-17457 Greifswald
GERMANY
Tel.: +49 (0)3834 86-6301
Fax: +49 (0)3834 86-6410
E-Mail: Jan.Daebritz@med.uni-greifswald.de

INDEX OF THE ELECTRONIC SUPPLEMENTARY MATERIAL

SUPPLEMENTARY TABLES 3

Supplementary Table S1 3

Supplementary Table S2 4

Supplementary Table S3 5

Supplementary Table S4 6

SUPPLEMENTARY FIGURES 7

Supplementary Figure S1 7

Supplementary Figure S2 8

Supplementary Figure S3 9

SUPPLEMENTARY DISCUSSION 10

Limits of detection 10

Overfitting in post-selection inference 10

Augmented-data and latent projection 10

Coarsening of categorical predictors 10

Prior sensitivity analysis 10

SUPPLEMENTARY REFERENCES 11

SUPPLEMENTARY TABLES

Supplementary Table S1. Overview of the candidate predictors for the statistical model.

**Demographic information**					
Age	Sex	IBD clinic	Patient identifier (group-level term)		
**Laboratory findings (blood)**					
C-reactive protein	Hematocrit	Hemoglobin	Mean corpuscular volume	Platelet count	White blood cell count
**Laboratory findings (stool)**					
Fecal calprotectin					
**Parameters of medical history**					
Abdominal pain	Abdominal pain at night	Activity limitation	General well-being	Nocturnal stool	Stool quantity per 24 hours
Stool blood	Stool consistency				
**Physical examination**					
Abdominal finding*<br>Pressure pain<br>Resistance	Body weight gain	Extraintestinal manifestation	Perianal disease		

* Pressure pain and resistance were analyzed individually and once together in the category abdominal finding (conspicuous if one of the two parameters was pathological in the examination).

Supplementary Table S2. Characteristics of the study cohort. Age, body weight, and body height refer to the first visit of each patient.

**General characteristics**	Patients (total: 59)
**Male, N (%)**	24 (40.7)
**Female, N (%)**	35 (59.3)
**Age, years**	M: 13.1 (± 3.4), Q0: 2.5, Q25: 11.0, Q50: 13.6, Q75: 15.6, Q100: 17.9
**Body weight, kg**	M: 48.2 (± 16.5), Q0: 14.7, Q25: 34.7, Q50: 49.0, Q75: 59.1, Q100: 83.0
**Body height, cm**	M: 154.9 (± 18.0), Q0: 88.0, Q25: 147.0, Q50: 157.5, Q75: 168.3, Q100: 187.0
**Histologic score, N (%)**	Visits (total: 91)
**Remission**	5 (5.5)
**Mild**	19 (20.9)
**Moderate**	52 (57.1)
**Severe**	15 (16.5)

Age, body weight and body height are presented as mean (M) ± standard deviation, minimum (Q0), 1st quartile (Q25), median (Q50), 3rd quartile (Q75), and maximum (Q100).

Supplementary Table S3. Categorical candidate predictors and their distribution across visits.

Name	Description
Abdominal pain	53 (58%) “Yes”, 37 (41%) “No”, 1 (1%) MVs
Abdominal pain at night	6 (7%) “Yes”, 84 (92%) “No”, 1 (1%) MVs
Abdominal finding	65 (71%) "Without pathological findings", 26 (29%) "With pathological findings"
Abdominal finding – pressure pain	23 (25%) “Yes”, 68 (75%) “No”
Abdominal finding – resistance	4 (4%) “Yes”, 87 (96%) “No”
Appetite[1]	52 (57%) "Good", 21 (23%) "Reduced", 5 (5%) "Poor", 13 (14%) MVs
Activity limitation	39 (43%) “Yes”, 51 (56%) “No”, 1 (1%) MVs
Extraintestinal manifestation[2]	6 (7%) “Yes”, 85 (93%) “No”
General well-being	53 (58%) "Very good, good", 38 (42%) "Reduced, poor, very poor"
Height gain[3,5]	83 (91%) “Yes”, 6 (7%) “No”, 2 (2%) MVs
IBD clinic	37 (41%) "IBD center A", 54 (59%) "IBD center B"
Nocturnal stool	22 (24%) “Yes”, 69 (76%) “No”
Perianal disease[4]	3 (3%) “Yes”, 88 (97%) “No”
Sex	54 (59%) "Female”, 37 (41%) “Male”
Stool blood	58 (64%) “Yes”, 33 (36%) “No”
Stool consistency	23 (25%) "Formed", 68 (75%) "Semi-formed or liquid”
Stool quantity	51 (56%) "≤ 3 stools per 24 hours", 40 (44%) "> 3 stools per 24 hours"
Weight gain[5]	63 (69%) "Weight gain, voluntary stable weight, voluntary weight loss", 26 (29%) "Involuntary stable weight, involuntary weight loss", 2 (2%) MVs

[1]excluded due to a high number of missing values (MVs); [2]"Yes" is defined as at least one of the following: fever ≥ 38.5 °C for more than three days, arthritis, uveitis, erythema nodosum, pyoderma gangrenosum; [3]excluded due to ambiguous or missing documentation; [4]"Yes" is defined as at least one of the following: rhagade, fissure, indolent/active fistula, abscess, multiple/inflamed tag, abscess, perianal eczema; [5]approx. 3-6 months before clinic visits.

Supplementary Table S4. Predictor ranking for histologic inflammation based on the projection-predictive feature selection (PPFS).

Submodel size	Log FC	Log platelet count	General well-being (category "Reduced, poor, very poor")
1	1	0	0
2	0	0.96	0
3	0	0.04	0.68

The order of the last 3 column names follows the PPFS's full-data predictor ranking. For each submodel size *m* (first column), the values from the last 3 columns give the proportions of cross-validation (CV) folds which have the predictor from the respective column at position *m* of their forward search's predictor ranking (there is one forward search per CV fold). Note that the proportions don't need to sum to 1 (neither row-wise nor column-wise) because the forward search was terminated at submodel size 3 (which is less than the number of predictor terms in the reference model). Apart from the patient ID (not shown here), all predictors were standardized (centered and scaled) prior to modeling. Here, "log" is the natural logarithm. Abbreviations: FC, fecal calprotectin.

SUPPLEMENTARY FIGURES

Supplementary Figure S1

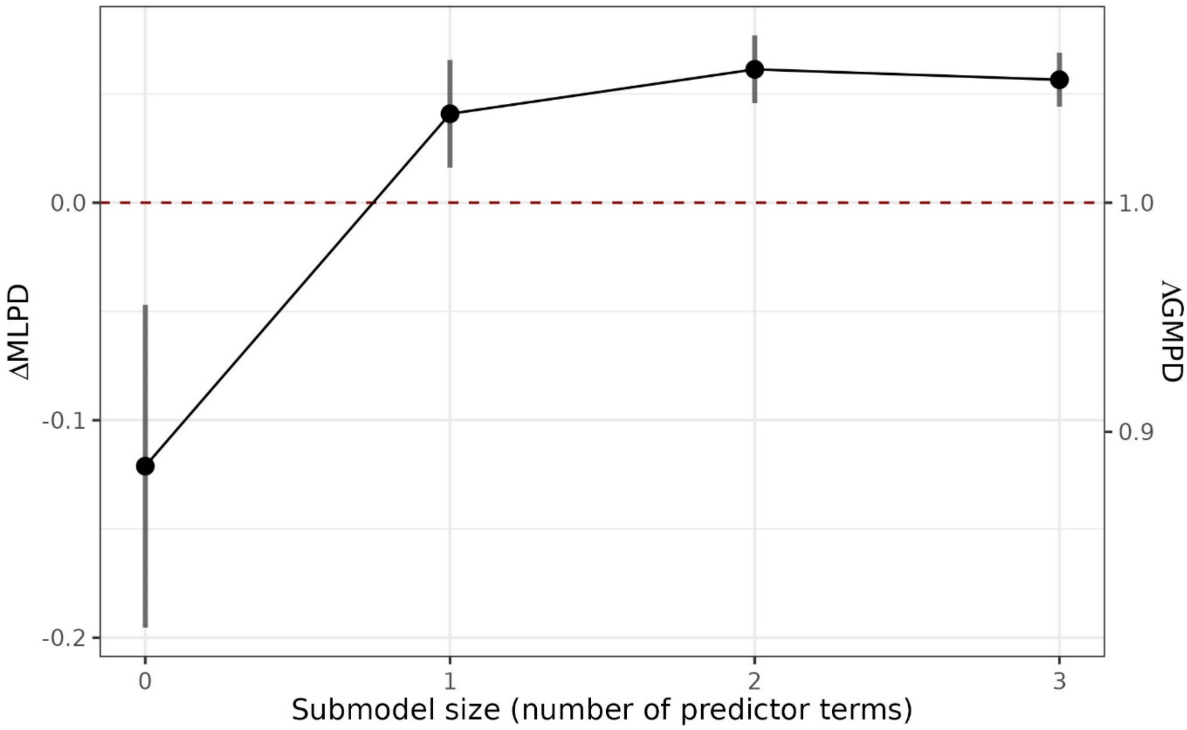

Model size selection plot from the projection-predictive feature selection (PPFS). This plot is based on the mean log predictive density (MLPD) as predictive performance measure on the left y-axis which, when exponentiated to the base of the natural logarithm, gives the geometric mean predictive density (GMPD) on the right y-axis. Here, the GMPD is the geometric mean of the predictive probabilities at the observed outcome categories. The higher the MLPD or the GMPD, the better the predictive performance. The x-axis shows the number of predictors during the forward search. The dashed red line indicates the reference model's predictive performance, which is here by definition 0 (on the left y-axis) and 1 (on the right y-axis) since on the left y-axis, the plot visualizes ΔMLPD, defined as the submodel MLPD minus the reference model MLPD (and on the right y-axis, the exponentiation gives ΛGMPD, the ratio of the submodel GMPD to the reference model GMPD). The uncertainty bars here indicate ± 1 standard error of the ΔMLPD estimator.

Supplementary Figure S2

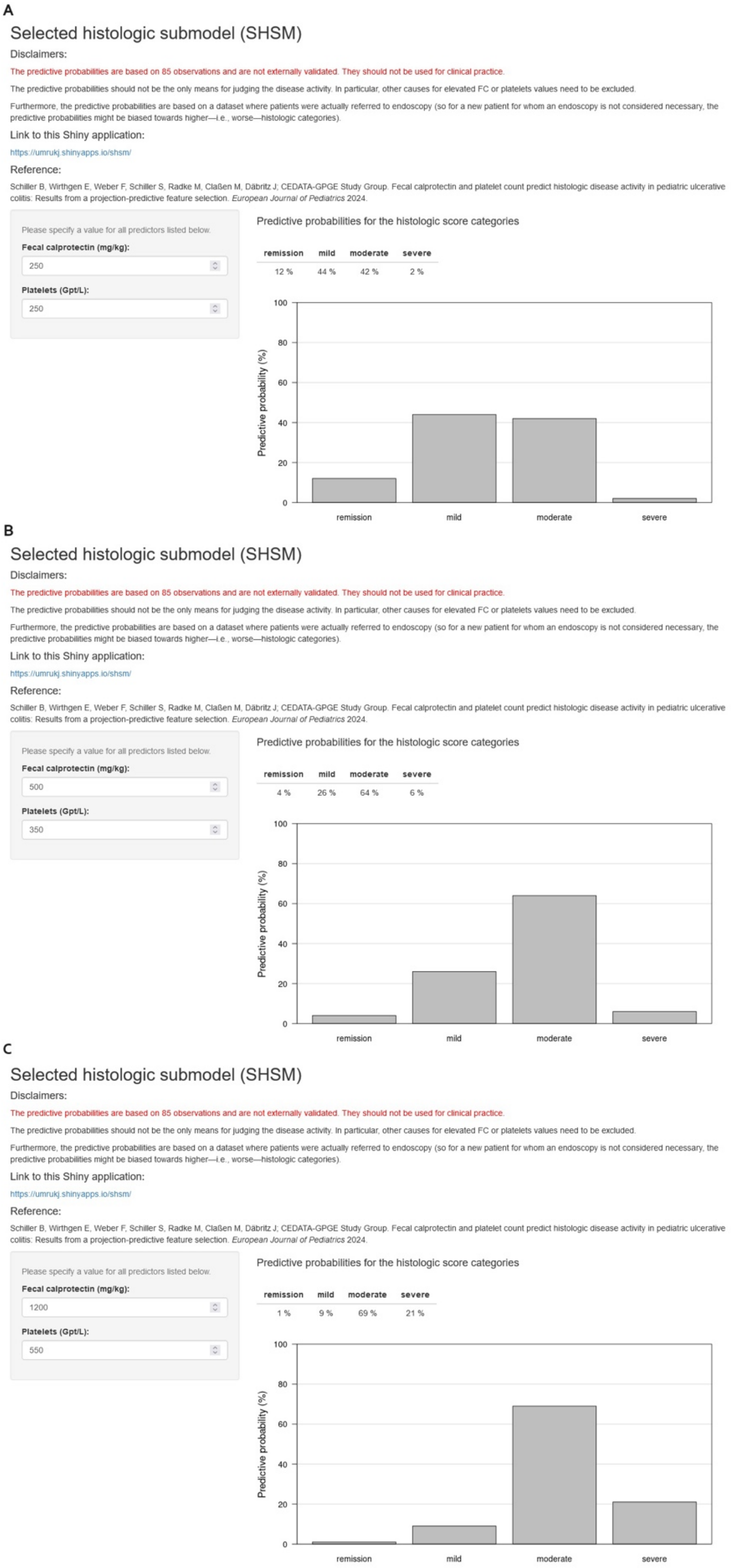

Screenshot of an example Shiny application. Predictor input fields are shown on the left-hand side and the returned predictive probabilities of histologic disease activity (based on the selected histologic submodel, SHSM) are shown on the right-hand side. The figure illustrates exemplarily the output for mildly (A), moderately (B) and severely (C) elevated values of FC and platelet count (https://umrukj.shinyapps.io/shsm/).

Supplementary Figure S3

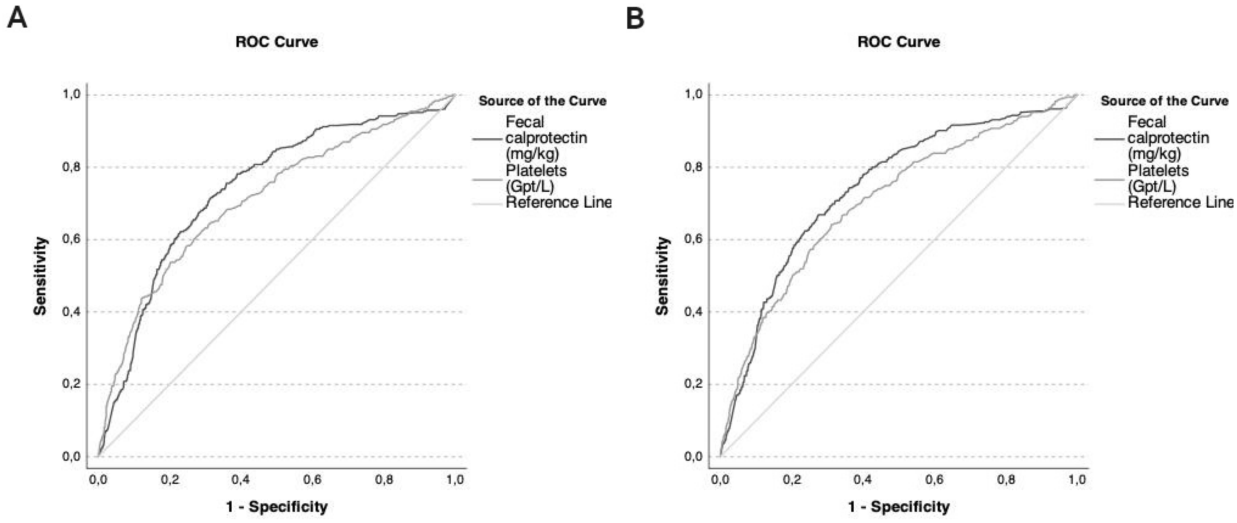

ROC curve analysis for platelets and fecal calprotectin to differentiate between mild and moderate and severe disease activity according to PGA (A) and PUCAI (B) using data from the CEDATA-GPGE registry.

SUPPLEMENTARY DISCUSSION

Limits of detection

Even though approved clinical laboratory parameters have a high validity, the statistical value of some laboratory parameters may be limited by analytical conditions such as limits of detection, resulting in so-called right- or left-censored data. In our study, the lower limit of detection for CRP was either 1.0 mg/L or 0.6 mg/L, depending on the study centers where the samples were measured. This resulted in the accumulation of multiple observations at these specific values. While we acknowledge the existence of advanced statistical techniques for handling censored predictors, incorporating such methods was beyond the scope of our investigation. However, these statistical limitations should be considered in future studies.

Overfitting in post-selection inference

The PPFS does not guard its post-selection inference (and hence our SHSM's predictions) against the overfitting induced by the selection of the final submodel size. However, this overfitting should not be severe because in our case, we select the final submodel size from only 4 different submodel sizes (0 to 3), see also Piironen and Vehtari [1] for the theoretical argument.

Augmented-data and latent projection

The augmented-data projection [2] constitutes the exact counterpart to the latent projection (the approximate approach proposed by Catalina et al. [3] employed here). However, for multilevel models as in our case, the augmented-data projection (as well as in some cases also the PPFS's traditional projection) needs further theoretical investigations [2] and hence has not been employed here.

Coarsening of categorical predictors

Originally, some categorical predictors had more than two categories but have been coarsened to two categories here. Where possible (some categorical predictors had to be coarsened due to rare categories), future research should investigate whether using the original (finer) categories would alter our results.

Prior sensitivity analysis

As recommended for Bayesian analyses [4], we conducted a prior sensitivity analysis to check how a different prior distribution (for the reference model) would have altered our results. To this end, we replaced the regularized horseshoe prior for the regression coefficients and brms's default prior for the standard deviation of the patient-specific group-level (or "random") effects by the R2D2M2 prior [5] with hyperparameters that imply more shrinkage than by default. Our results were only slightly affected; in particular, the selected predictors would still have been FC and platelet count. Thus, we consider our chosen prior to be sufficiently robust (in this regard). We

note, however, that we have not altered brms's default prior distribution for the latent thresholds (i.e., for the model's “intercepts”). Details may be found in the source code (see data availability statement).

SUPPLEMENTARY REFERENCES

1. Piironen J, Vehtari A (2017) Comparison of Bayesian predictive methods for model selection. Stat Comput 27:711–735. doi:10.1007/s11222-016-9649-y
2. Weber F, Glass Ä, Vehtari A (2023) Projection predictive variable selection for discrete response families with finite support. arXiv preprint. Available at: https://doi.org/10.48550/arXiv.2301.01660
3. Catalina A, Bürkner PC, Vehtari A (2021) Latent space projection predictive inference. arXiv preprint. Available at: https://doi.org/10.48550/arXiv.2109.04702
4. Depaoli S, van de Schoot R (2017) Improving transparency and replication in Bayesian statistics: The WAMBS-Checklist. Psychol Methods 22:240–261. https://doi.org/10.1037/met0000065
5. Aguilar JE, Bürkner PC (2023) Intuitive joint priors for Bayesian linear multilevel models: The R2D2M2 prior. Electron J Statist 17:1711–1767. https://doi.org/10.1214/23-EJS2136
